# Profound seasonal changes in brain size and architecture in the common shrew

**DOI:** 10.1007/s00429-018-1666-5

**Published:** 2018-04-16

**Authors:** Javier Lázaro, Moritz Hertel, Chet C. Sherwood, Marion Muturi, Dina K. N. Dechmann

**Affiliations:** 1 Department of Migration and Immuno-Ecology, Max Planck Institute for Ornithology, 78315 Radolfzell, Germany; 2 Department of Biology, University of Konstanz, 78457 Konstanz, Germany; 3 Department of Behavioural Neurobiology, Max Planck Institute for Ornithology, 82319 Seewiesen, Germany; 4 Department of Anthropology, The George Washington University, 20052 Washington, DC USA

**Keywords:** Brain mass, Tissue regeneration, Neuron atrophy, Dendritic plasticity, Brain anatomy, Dehnel’s phenomenon

## Abstract

The seasonal changes in brain size of some shrews represent the most drastic reversible transformation in the mammalian central nervous system known to date. Brain mass decreases 10–26% from summer to winter and regrows 9–16% in spring, but the underlying structural changes at the cellular level are not yet understood. Here, we describe the volumetric differences in brain structures between seasons and sexes of the common shrew (*Sorex araneus*) in detail, confirming that changes in different brain regions vary in the magnitude of change. Notably, shrews show a decrease in hypothalamus, thalamus, and hippocampal volume and later regrowth in spring, whereas neocortex and striatum volumes decrease in winter and do not recover in size. For some regions, males and females showed different patterns of seasonal change from each other. We also analyzed the underlying changes in neuron morphology. We observed a general decrease in soma size and total dendrite volume in the caudoputamen and anterior cingulate cortex. This neuronal retraction may partially explain the overall tissue shrinkage in winter. While not sufficient to explain the entire seasonal process, it represents a first step toward understanding the mechanisms beneath this remarkable phenomenon.

**Electronic supplementary material:**

The online version of this article (10.1007/s00429-018-1666-5) contains supplementary material, which is available to authorized users.

## Introduction

An animal’s brain structure and size arise from developmental mechanisms that are shaped by socioecological adaptations and life history (Striedter 2005). The brain functions related to behavior and cognitive processes, in particular, provide individuals with the capacity to adapt to environmental changes over the life span. But maintenance and function of brain tissue require large amounts of energy, using up a substantial proportion of individuals’ metabolic budget (Aiello and Wheeler 1995; Laughlin et al. 1998; Niven and Laughlin 2008). Therefore, the actual size of an animal’s brain and each of its constituent parts is likely the result of a trade-off between the advantages of higher computational capacity and the costs of energetic demands. However, the physiological and cellular mechanisms that lead to an optimal neural structure remain unclear (Bullmore and Sporns 2012).

Of particular interest for understanding these adaptive and energetic trade-offs are species exhibiting large variation of brain size between individuals or over the life span. Habitat seasonality provides predictable fluctuations in the environment and resource availability, which impact energy budget allocation. Consequently, seasonal changes in physiology and behavior of animals are often observed (but see Bolhuis and Macphail 2001). The best known examples of seasonal brain plasticity are found in some songbirds, where the size of song control nuclei changes in anticipation to the breeding season (Nottebohm 1981; Brenowitz et al. 1991; Smith et al. 1997; Tramontin et al. 1998). For example, in canaries the volume of the HVC increases by 50% from fall to spring, and the robust nucleus of the arcopallium (RA) increases by 43%, leading to a change in overall brain mass of 13% (Nottebohm 1981). In food hoarding black-capped chickadees, the hippocampus decreases by 17% during the non-hoarding season, when the spatial cognitive demands of food caching are reduced (Krebs et al. 1989; Barnea and Nottebohm 1994, 1996; Smulders et al. 1995; Bartkowska et al. 2008). The volumetric changes in the avian HVC and hippocampus have been associated with changes in cell numbers (Tramontin et al. 1998; Smulders et al. 2000); but changes in the RA are based on neuron size and spacing, as well as an increase in dendritic trees (Smith et al. 1997; Tramontin and Brenowitz 2000). Similar processes can also be found in food-hoarding mammals. The males of Richardson’s ground squirrel store food before hibernation, and hippocampal size increases by 15% during that period (Burger et al. 2013). Similarly, hippocampus volume increases during the caching period in gray squirrels (Lavenex et al. 2000b) and chipmunks (Barker et al. 2003). In contrast, fluctuations in hippocampal volume of mammals do not appear to be correlated with changes in total cell numbers (Lavenex et al. 2000a; Barker et al. 2003, 2005). Notably, during hibernation ground squirrels exhibit a reversible decrease in hippocampal dendrite arbors, as well as in the number and size of dendritic spines (Popov and Bocharova 1992; Popov et al. 1992), suggesting that dendritic and synaptic plasticity are important mechanisms underlying the volumetric reorganization. Seasonal brain changes in other mammals, including humans (Hofman and Swaab 1992), are restricted to the microstructure and biochemistry of hypothalamic (Hofman and Swaab 2002) and hippocampal regions (Magariños et al. 2006; Workman et al. 2009).

To learn more about the structural basis of adaptive brain size variability, we investigated the most extreme known case of individual seasonal variation in mammalian brain size and architecture. The brains of some species of red-toothed shrews (*Sorex* spp.) decrease in mass from summer to winter by 20% or more, followed by regrowth of ca. 15% (i.e., Dehnel’s Phenomenon; Bielak and Pucek 1960; Pucek 1965a; Yaskin 1994; Bartkowska et al. 2008). These changes are accompanied by correlated variation in braincase size (Dehnel 1949; Serafinski 1955; Caboń 1956; Bielak and Pucek 1960; Taylor et al. 2013; Lázaro et al. 2017), as well as the size of internal organs including the spleen and liver (Pucek 1965b), and the length of the spine (Hyvarinen 1969). The change in overall brain size dramatically affects brain architecture across seasons; in a Russian population of shrews, neocortex and hippocampus show the most profound winter decrease compared to other brain regions, while other regions remain stable in size or grow in the spring (Yaskin 1994). In parallel, cognitive skills also exhibit seasonal variability, with small-brained winter shrews showing lower spatial learning skills than both large-brained summer juveniles and spring adults (Lázaro et al. 2018). This result is congruent with winter decrease in the hippocampus and neocortex, as these regions process information on spatial cognition, cue sensitivity and memory. Similar to food-storing birds and ground squirrels, the changes in shrew brain and behavior have been attributed to different space use across the seasons (Lázaro et al. 2018). Individual territories are smaller during winter, but then expand during the breeding season in spring and summer (Stockley and Searle 1998; Yaskin 2005).

In this study we examined neuroanatomical reorganization underlying seasonal changes in brain size of a red-toothed shrew species, the common shrew (*Sorex araneus*). We first confirmed that overall brain size changes at our study site in southern Germany and measured the volumes of brain regions over the 1-year life span from individuals collected at this location. This was important as seasonal changes may vary in their extent based on the severity of local conditions and previous studies were carried out at higher latitudes (Pucek 1970). We therefore predicted a less pronounced seasonal change in our study population than in the northern populations. In addition, we analyzed the magnitude of change in each brain region. Since these differences might be driven by the changes in cognitive demands along the seasons, we expected to find a more intense winter decrease in regions with diminished functions during that period such as hippocampal and cortical areas. Furthermore, we expected that if overall brain size regrowth in spring is driven by territory expansion in preparation for reproduction, then sex differences would be evident, especially in the hippocampus as the expansion of territories in spring is more intense in males than in females (Stockley et al. 1994; Rychlik 1998; Stockley and Searle 1998; Yaskin 2005). Also, an important determinant for the differences between regional changes might be their differential flexibility. Ontogenetic timing can be a predictor of evolvability and plasticity (Finlay and Darlington 1995; Clancy et al. 2001); thus, we expected latter developed regions such as neo-, rhinal and piriform cortices to undergo more drastic changes. However, if the seasonal change is driven by a purely energetic constraint in winter, we then predict observing the least drastic winter decline in the least costly regions, i.e., thalamic regions, minimizing energy demands during the period of resource scarcity.

We assessed further whether the mechanisms of size variation were identifiable at the cellular level. A previous study found no evidence for changes in cell numbers in the hippocampus of shrews over the life span (Bartkowska et al. 2008). Thus, we predicted that size changes would be driven by differences in neuron morphology. To test this, we traced Golgi-impregnated neurons in several selected brain regions. At the cellular level, we expected to observe changes in the soma and/or dendritic morphology of seasonally changing regions that correlate with the magnitude of the change in the specific brain region.

This study makes an important contribution to understanding the link between brain size and the underlying anatomical structures in this unique mammalian species where such pronounced brain size variability occurs predictably within individuals over their life span.

## Methods

### Trapping and processing of specimens

Trapping took place monthly between August 2013 and October 2015 in Möggingen, Germany (longitude 8.994, latitude 47.766). Shrews were trapped with wooden live traps (PPUH A. Marcinkiewicz, Rajgród, Poland) baited with mealworms and checked at 2-h intervals. Once caught, we brought the shrews to the laboratory, where we perfused them transcardially with phosphate-buffered saline (PBS) followed by freshly prepared 4% formaldehyde in PBS under deep isoflurane anesthesia. We immediately extracted the brains from the skull, separated the hemispheres, and weighed them to the nearest 0.001 g before postfixation for 2 weeks in 4% buffered paraformaldehyde. We then transferred the tissue to PBS/0.1% sodium azide at 4 °C for long-term storage. The right hemisphere was used to reconstruct brain region volumes; the left hemisphere was used for Golgi staining and neuron morphology analyses.

### Age and sex determination

Based on the time of the year and the degree of gonadal development (Churchfield 1990), we classified individuals into three age groups: summer juvenile (sexually immature, from June–September); winter subadult (sexually immature, from December–March); and spring–summer adult (sexually mature, from May–August). Because *S. araneus* has a maximum life span of ~ 18 months, there is no generation overlap of mature adults. During the very brief period of overlap between summer juveniles and adults, they can be easily distinguished by the degree of development of the gonads (Churchfield 1990).

To determine the sex of immature individuals (all individuals until the spring following the year of birth), we used a PCR-based gonosomal sexing method (Roos, DPZ Göttingen, unpublished). DNA was extracted from tail tip samples using standard DNeasy kits (Qiagen, GmbH, Hilden).

### Calculation of brain region volumes

**Fig. 1:**
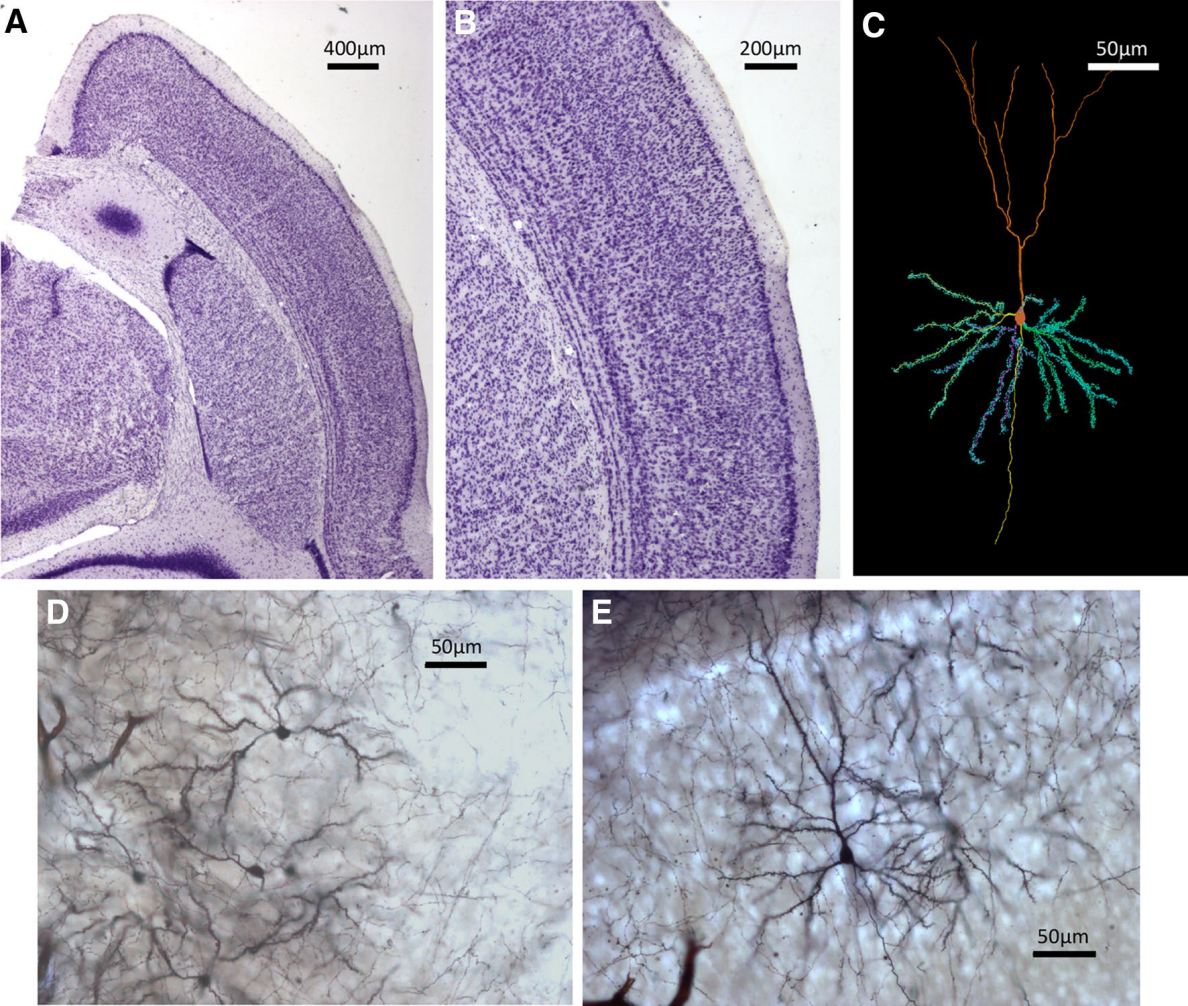
Examples of histological sections and neuron tracings. **a** Nissl-stained section of a shrew brain hemisphere showing a dorsal medial area. **b** Details of a Nissl-stained section depicting a portion of the neocortex. **c** Neuron tracing as depicted by Neurolucida. **d** Medium spiny neurons in the caudoputamen stained with the Golgi technique. **e** Pyramidal neurons in the neocortex

**Fig. 2:**
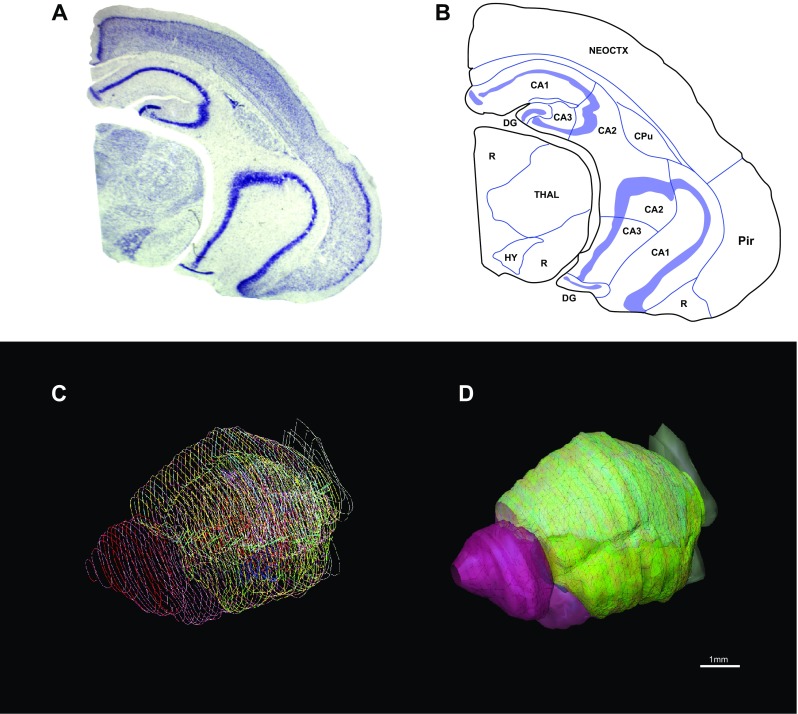
**a** Coronal section of a hemisphere stained with Nissl. **b** Outlines of the brain regions traced on a Nissl-stained section using Neurolucida software. *NEOCTX* neocortex, *Pir* piriform cortex, *CPu* caudoputamen, *DG* dentate gyrus, *THAL* thalamus, *HY* hypothalamus, *R* rest of brain. **c** Stack of all section outlines of a hemisphere. **d** 3D reconstruction of an hemisphere based on section outlines

We quantified the volume of brain structure from ten individuals (five males and five females) of each age group (*N* = 30). Before sectioning, the left hemisphere was immersed in a series of 10, 20 and 30% sucrose in PBS for cryoprotection. We cut the tissue on a freezing sliding microtome (Reichert-Jung Hn-40) to obtain 30 µm-thick coronal sections. We mounted every fifth section on slides and stained them with 0.5% cresyl violet (Figs. 1, 2). We examined the following brain regions: olfactory bulb, neocortex, rhinal and piriform cortices, caudoputamen, amygdala, nucleus accumbens, thalamus, hypothalamus, hippocampus, dentate gyrus, CA1, CA2, CA3, subiculum and cerebellum and the total hemisphere. We located and defined these brain regions based on cytoarchitectural descriptions from insectivores (Catania et al. 1999; Catania 2000; Naumann et al. 2012). As a reference, we also used a mouse brain atlas (Paxinos and Franklin 2013). We used an Olympus BX51 microscope under an Olympus UIS2 Plan N 2× (NA = 0.02) dry objective interfaced with a Neurolucida software system (MBF Bioscience, Williston, VT, USA) to outline each brain region (Fig. 2). The system utilized a MicroFire Digital CCD 2 Megapixel camera (Optronics, Goleta, CA, USA) and an HP Z27i monitor with 2560 × 1440 resolution. The Cavalieri principle was used to calculate the volume of each region from the sum of brain region areas measured in each section multiplied by the interval distance and section thickness. Volumes were automatically calculated in the software extension Neurolucida Explorer.

A correction factor was used for each individual to account for the shrinkage occurring during the histological processing of the tissue (de Sousa et al. 2010). The correction factor for each brain was calculated as the quotient between the freshly extracted hemisphere volume and the final volume of that whole hemisphere derived from the measurement of outlined slides. The fresh hemisphere volume was calculated by dividing the fresh hemisphere mass by the specific gravity of brain tissue (1.036 g/cm^3^; Stephan 1960). Each brain-specific correction factor was then applied to the brain region volumes for that specimen.

The final volumes obtained for each brain region as well as whole hemispheres were size corrected dividing by the upper tooth row, a metric which is stable across seasons (Lázaro et al. 2017). All tracings were done blind by a single observer (MM).

### Neuron tracing and quantification

**Fig. 3:**
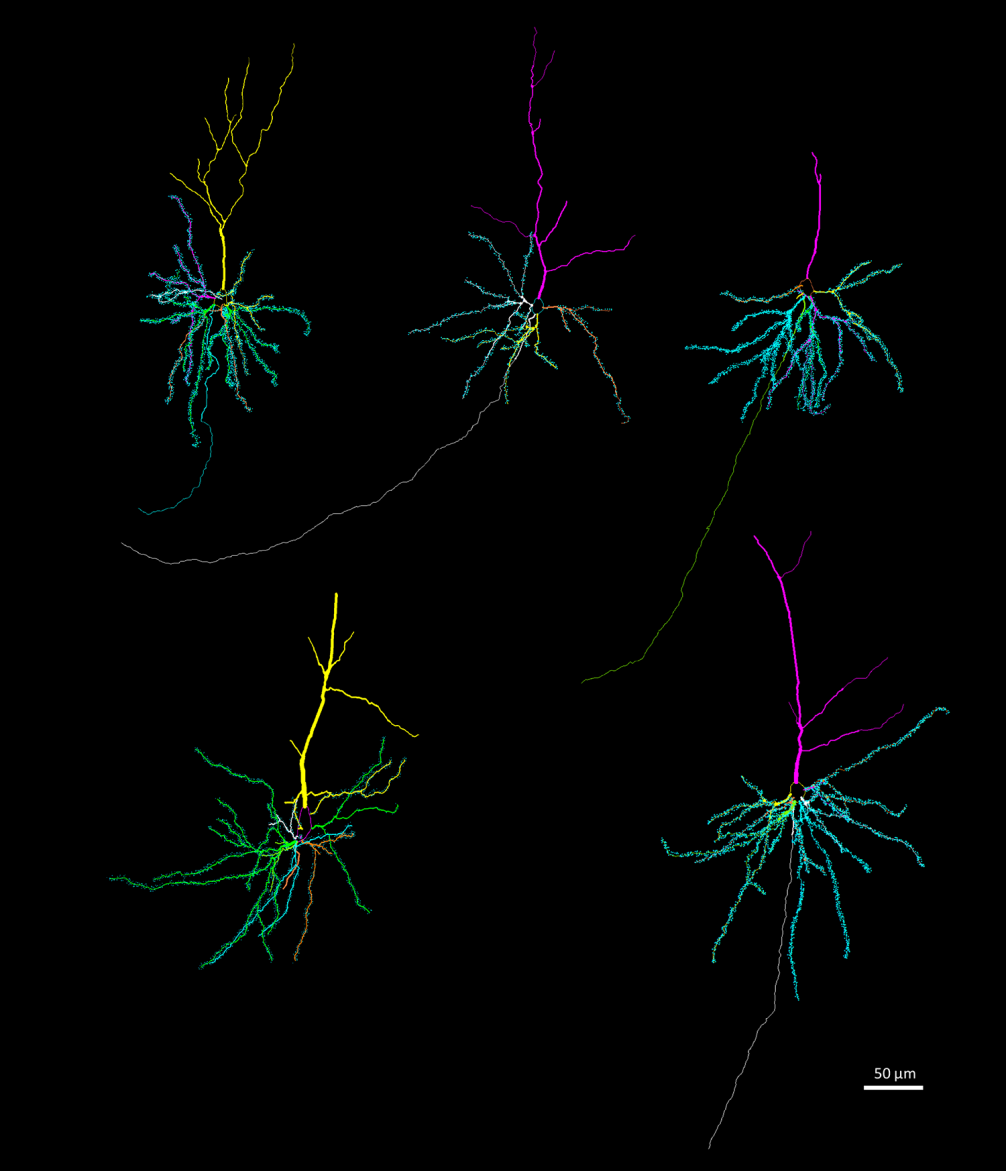
Examples of reconstructions of pyramidal neurons in the anterior cingulate cortex. Dendritic spines are indicated in the basal dendrites. Reconstructions were done in Neurolucida

**Fig. 4:**
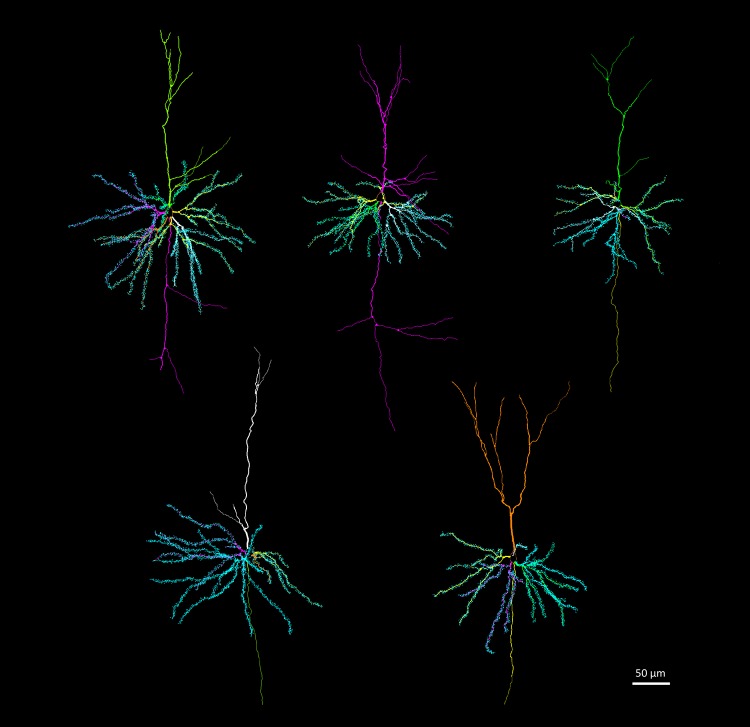
Examples of reconstructions of pyramidal neurons in the somatosensory cortex. Dendritic spines are indicated in the basal dendrites. Reconstructions were done in Neurolucida

**Fig. 5:**
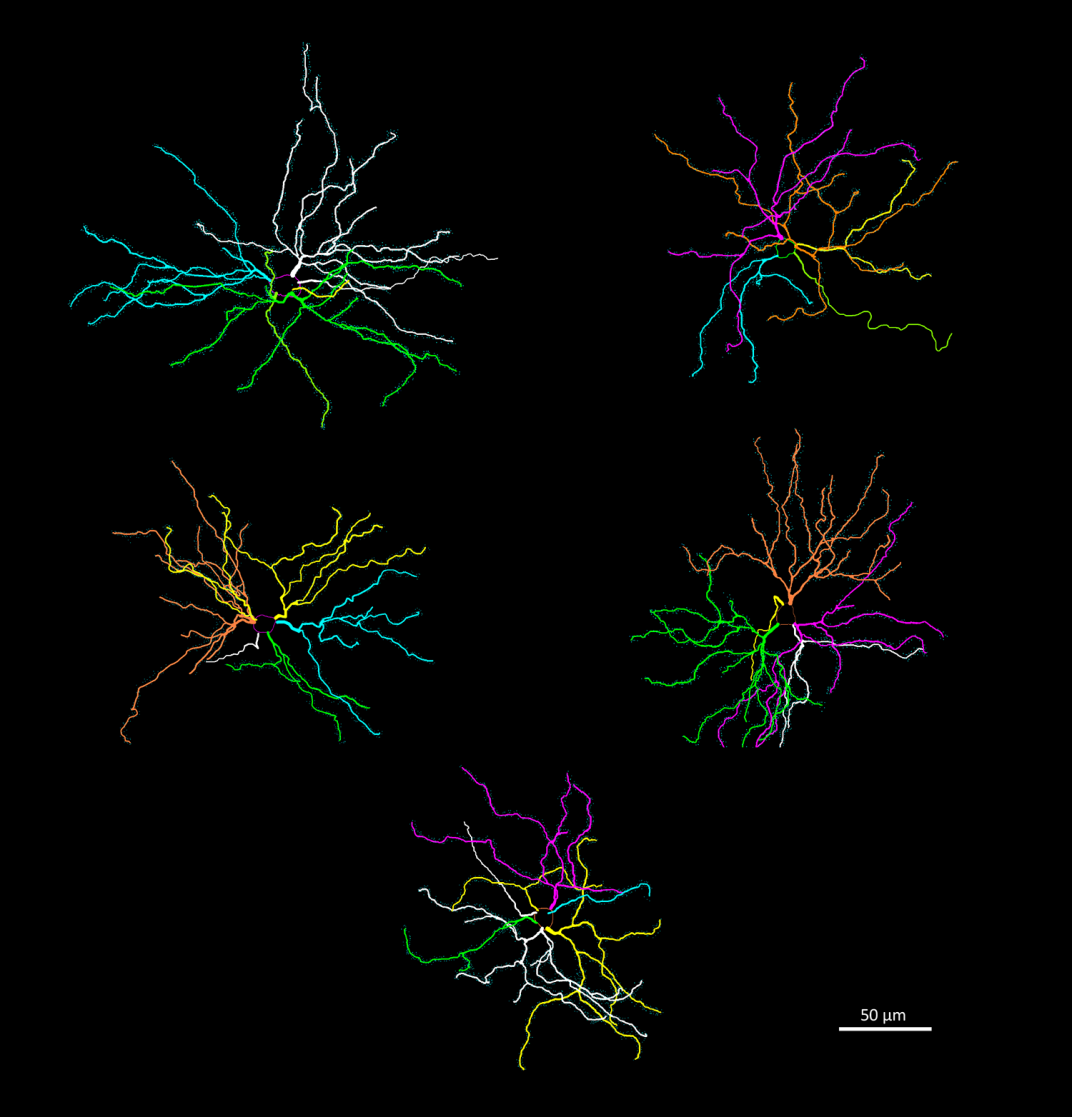
Examples of reconstructions of spiny neurons in the caudoputamen. Reconstructions were done in Neurolucida

We used brains from five males of each of the three age groups (*N* = 15) to study neuron architecture. Right hemispheres were processed by a modified rapid Golgi technique (Scheibel and Scheibel 1978) (Fig. 1). After processing, they were cut coronally in two halves, and both tissue blocks were serially sectioned at 100 µm with a Vibratome. We focused on three types of neurons: pyramidal neurons of layer III–IV in the anterior cingulate cortex (Fig. 3) and in the somatosensory cortex (Fig. 4); and medium spiny neurons of the caudoputamen (Fig. 5). The brain regions were identified based on cytoarchitectural criteria and using the Nissl-stained sections as reference. The Golgi technique only stains a limited number of neurons randomly, which allows visualizing separated cells and their processes (Scheibel and Scheibel 1978). We selected neurons that appeared fully impregnated, isolated from other stained neurons, with their soma centered within the section thickness and which had as complete dendritic trees as possible (Fig. 1).

We traced 25 neurons from each brain region per age group, randomly chosen—within the above criteria—among the five individuals in each age group (total = 225 neurons, 75 neurons of each neuron type). To avoid any possible bias, all neuron tracings were obtained by a single observer (JL) and performed blind to the individual and age group. Neurons were quantified along *x*-, *y*-, and *z*-coordinates using the Neurolucida system (see above) under an Olympus UIS2 Plan N 100× (NA = 1.25) oil objective. For each neuron, the soma was traced in the widest two-dimensional point to obtain its cross-sectional area and the dendritic tree was traced accounting for dendritic diameter, marking all bifurcations and quantifying all visible spines, without determining spine type. Incomplete dendrites that were cut in the section edge were not followed into the adjacent section and were marked as incomplete endings. We quantified the following metrics: soma size (area), total dendritic length and volume, spine number and density. All metrics were automatically extracted in Neurolucida Explorer. For the cortical neurons, only the basal dendrites were examined because often the apical dendrites were incomplete.

### Data analyses

We analyzed the volumes of entire hemispheres using ANCOVA with age group, sex, and their interaction as explanatory variables. We used a linear mixed effects model to analyze the volumes of all brain regions, with age group, brain region and sex and their interactions as factors, and individual as random effect. To analyze the data from the neuron tracings, we used a linear mixed-effects model for all metrics (soma size, dendritic length, dendritic volume, number of spines and spine density), which were used as dependent variables, age group as factor, and individual was included as random effect. We analyzed each region (anterior cingulate cortex, somatosensory cortex and caudoputamen) separately.

To quantify the differences between age groups, and between age groups and sexes, we estimated probabilities of the differences (*P*) and 95% Bayesian credible intervals (CrI). We used Monte Carlo simulations to obtain 20,000 random values from the joint posterior distribution of the model parameters assuming flat prior distributions. We calculated 95% CrI as the 2.5 and 97.5% quantiles of the marginal posterior distributions of the parameters.

For the linear mixed models, we used the function lmer from the R package lme4 (Bates et al. 2014). For Monte Carlo simulations we used the function sim from the R package arm (Gelman and Su 2015). All analyses were performed in R 3.3.1. (R Core Team 2016).

## Results

### Brain region volumes

**Fig. 6:**
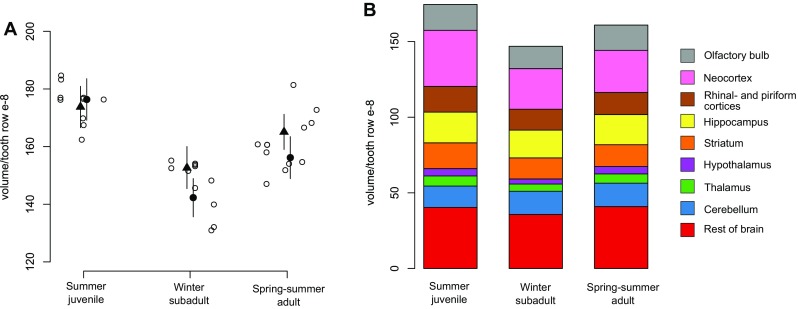
Seasonal changes in volume of brain and brain regions. **a** Volume of the entire brain hemisphere during the three age stages of the cycle, corrected by tooth row. Open circles represent individuals, triangles (males) and closed circles (females) are the means of each cycle stage, and bars are credible intervals. **b** Corrected volume for the entire brain and each brain region in the three stages. The area of each colored section represents the mean. See Table 1 for detailed results

**Fig. 7:**
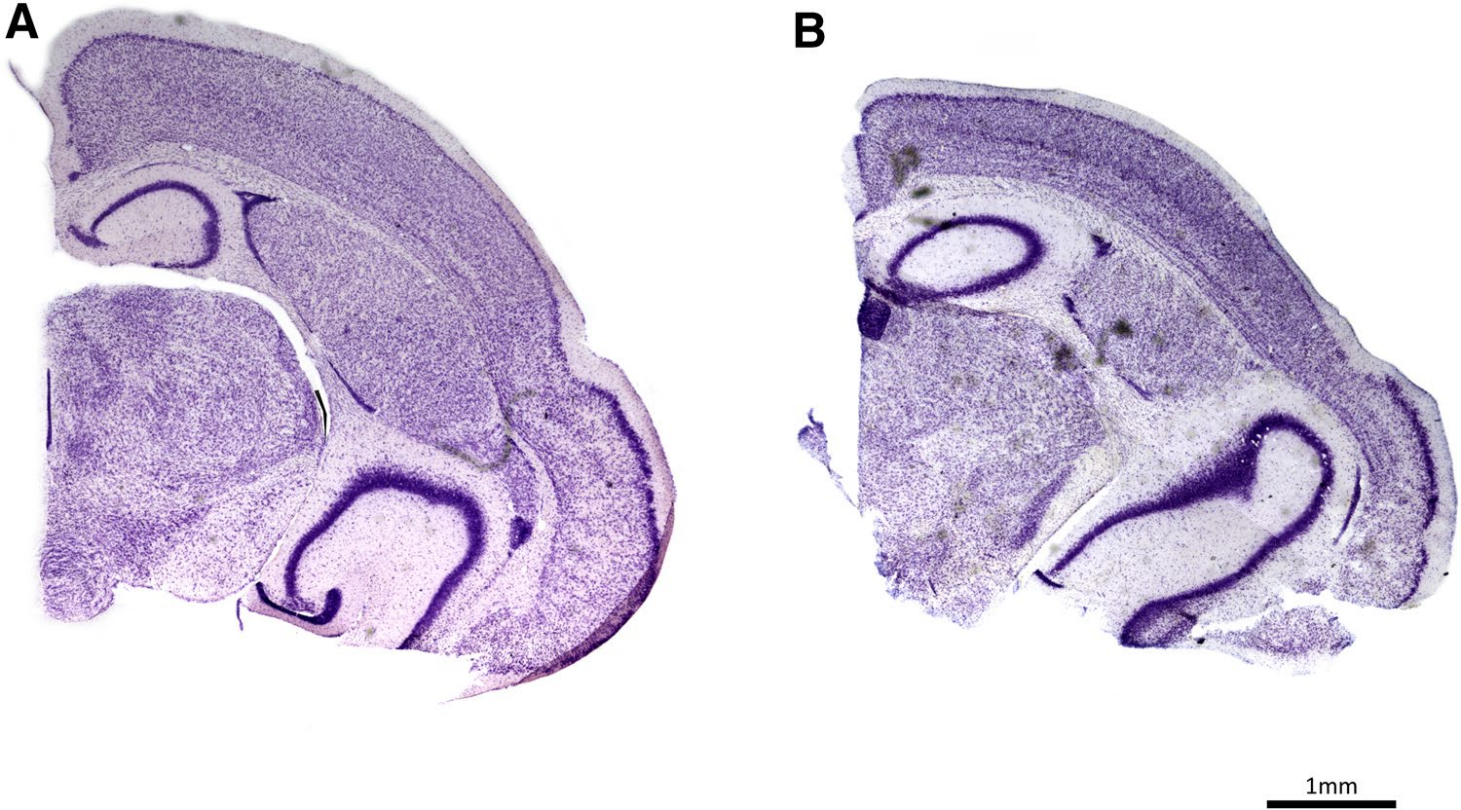
Exemplary brain coronal sections cut at a similar level in a summer juvenile (**a**) and a winter subadult (**b**) and depicted at the same scale

Total hemisphere volumes decreased on average by 16.1% from summer juveniles to winter subadults, and increased again by 9.8% from winter subadults to spring–summer adults (Fig. 6a, see absolute values in Online Resource Table S1 and a comparative example in Fig. 7). The winter decrease was more pronounced in females than males, which led to females with smaller brains during winter. The spring regrowth was similar for both sexes and thus adult females’ brains remained smaller than males among adults (Fig. 2a; Table 1).

**Table 1:** Corrected volumes for all brain regions during the three age stages, mean differences between stages and probabilities (*P*) of the difference from summer juveniles to winter subadults (s–w) and from winter subadults to spring–summer adults (w–a) as calculated from the posterior distributions

	Summer juvenile		Winter subadult		Spring–summer adult		Difference summer–winter	*P*(s–w)	Difference winter–adult	*P*(w–a)
	Mean ± SD	95% CrI	Mean ± SD	95% CrI	Mean ± SD	95% CrI				
Brain hemisphere	175.1 ± 6.8	169.6/180.6	147.0 ± 8.9	141.7/152.3	161.3 ± 9.6	156.4/166.4	− 28.1 (− 16.1%)	> 0.99	14.3 (9.8%)	> 0.99
Males	173.8 ± 6.4	166.6/181.1	152.6 ± 2.7	145.3/159.8	165.1 ± 9.5	158.9/171.3	− 21.2 (− 12.8%)	> 0.99	12.5 (8.2%)	> 0.99
Females	176.4 ± 7.7	169.1/183.6	142.3 ± 9.7	135.7/148.9	156.2 ± 7.7	149.0/163.3	− 34.1 (− 19.3%)	> 0.99	13.9 (9.8%)	> 0.99
Rest of brain	40.3 ± 3.7	38.8/41.9	35.7 ± 5.3	34.3/37.2	40.9 ± 3.6	39.5/42.3	− 4.6 (− 11.5%)	0.99	5.2 (14.5%)	> 0.99
Males	41.1 ± 3.2	38.9/43.1	36.1 ± 1.7	34.0/38.2	43.1 ± 2.1	41.3/44.9	− 5.0 (− 12.1%)	0.97	7.0 (19.4%)	> 0.99
Females	39.6 ± 4.5	37.5/41.8	35.4 ± 7.4	33.5/37.3	37.8 ± 3.0	35.7/39.9	− 4.2 (− 10.6%)	0.95	2.4 (6.8%)	0.83
Cerebellum	14.2 ± 3.5	12.7/15.8	15.3 ± 3.7	13.9/16.8	15.5 ± 3.0	14.1/16.9	1.1 (8.0%)	0.22	0.2 (1.1%)	0.55
Males	15.6 ± 1.6	13.5/17.7	18.6 ± 2.3	16.5/20.7	14.7 ± 3.3	12.9/16.4	3.0 (19.0%)	0.05	− 3.9 (− 21.0%)	0.01
Females	12.7 ± 4.4	10.7/14.8	12.6 ± 1.7	10.7/14.5	16.6 ± 2.3	14.5/18.7	− 0.2 (− 1.2%)	0.54	4.0 (31.9%)	0.99
Thalamus	6.7 ± 1.5	5.1/8.2	4.8 ± 0.7	3.3/6.3	6.1 ± 1.0	4.7/7.5	− 1.9 (− 27.9%)	> 0.99	1.3 (27.5%)	> 0.99
Hypothalamus	4.9 ± 1.8	3.3/6.4	3.3 ± 2.3	1.8 /4.8	4.9 ± 2.0	3.5/6.3	− 1.5 (− 31.6%)	> 0.99	1.6 (47.8%)	> 0.99
Striatum	17.0 ± 2.3	15.5/18.6	13.9 ± 1.2	12.5/15.4	14.4 ± 1.8	13.0/15.8	− 3.1 (− 18.2%)	> 0.99	0.5 (3.5%)	0.74
Hippocampus	20.3 ± 1.8	18.8/21.9	18.4 ± 2.3	16.9/19.9	20.0 ± 2.0	18.5/21.4	− 1.9 (− 9.5%)	0.98	1.5 (8.4%)	0.96
Rhinal and piriform c	17.0 ± 1.8	15.4/18.5	13.8 ± 0.9	12.3/15.3	14.5 ± 1.7	13.1/16.0	− 3.2 (− 18.7%)	> 0.99	0.8 (5.4%)	0.88
Neocortex	37.1 ± 3.0	35.6/38.7	26.9 ± 1.8	25.4/28.3	27.9 ± 3.9	26.5/29.3	− 10.3 (− 27.7%)	> 0.99	1.0 (3.7%)	0.78
Olfactory b	17.1 ± 3.0	15.6/18.6	14.8 ± 1.4	13.3/16.2	16.8 ± 2.2	15.3/18.2	− 2.3 (− 13.7%)	0.99	2.0 (13.6%)	0.98
Males	15.8 ± 2.4	13.7/17.9	15.9 ± 0.8	13.4/18.1	17.9 ± 2.1	16.1/19.6	0.1 (0.7%)	0.47	1.9 (12.1%)	0.95
Females	18.4 ± 3.3	16.3/20.5	13.8 ± 1.1	11.9/15.7	15.2 ± 1.1	13.1/17.3	− 4.6 (− 25.1%)	> 0.99	1.4 (10.4%)	0.88

The values of each sex are depicted only for the regions where differences were found. The values are volumes divided by tooth row (µm^3^/ mm)

**Fig. 8:**
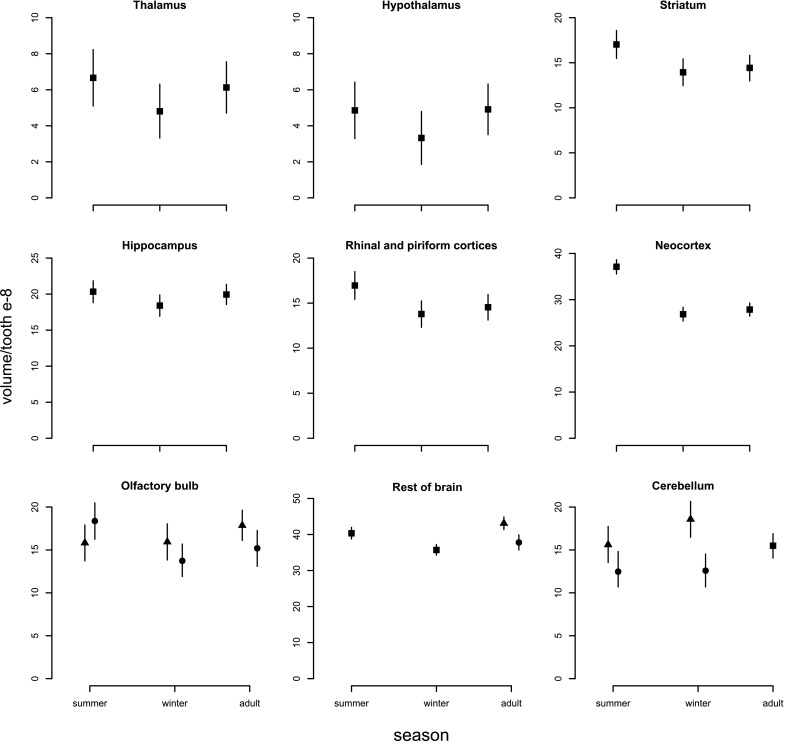
Seasonal changes in the volume of brain regions. Means at each stage and, whenever different, sex are represented by triangles (males) and closed circles (females). The mean of both sexes is depicted (squares) at the stages with no difference between sexes. Bars are credible intervals

**Fig. 9:**
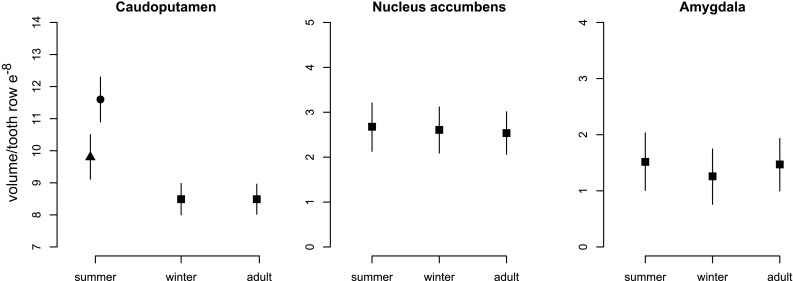
Seasonal changes in the volume of striatal subregions. Means at each stage and sex are represented by triangles (males) and closed circles (females). The mean of both sexes is depicted (squares) at the stages with no difference between sexes. Bars are credible intervals

**Fig. 10:**
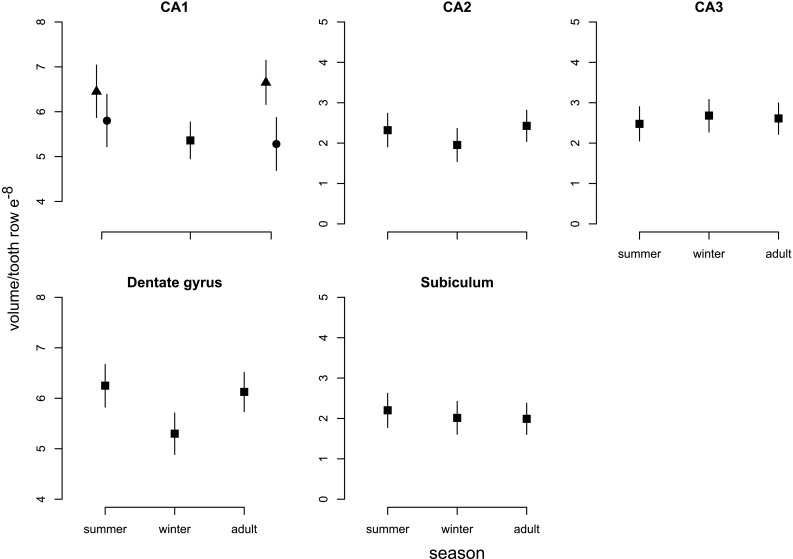
Seasonal changes in the volume of hippocampal subregions. Means at each stage and sex are represented by triangles (males) and closed circles (females). The mean of both sexes is depicted (squares) at the stages with no difference between sexes. Bars are credible intervals

When comparing brain region volumes among the age classes with Bayesian statistics, there was significant variation, with some regions undergoing more intense changes than others (Figs. 6b, 8; Table 1). The region showing the most intense changes in both directions was the hypothalamus (− 31.6%/+47.8%, respectively), followed by the thalamus (− 27.9%/+27.5%). Striatum volume decreased strongly from summer to winter by 18.2%, but did not regrow in spring. This decrease was mainly explained by a decrease in the caudoputamen by 20.7%, but no statistically obvious changes in nucleus accumbens and amygdala (Fig. 9, Online Resource Table S2). The hippocampus contributed to both winter decrease and spring regrowth in volume (− 9.5%/+8.4%), although we found different patterns between hippocampal subregions (Fig. 10, Online Resource Table S3): CA2 (− 15.8%/+24.2%) and dentate gyrus (− 15.2%/+15.6%) displayed marked changes in volume, while the change in CA1 was less pronounced (− 12.6%/+13.5%) and CA3 and subiculum did not change. Both sexes underwent a pronounced decline in CA1 from summer to winter, but only the CA1 of males regrew in spring by 18.4%, leading to sexual dimorphism in adult CA1. The large neocortex showed one of the most intense proportional decreases (− 27.7%) and thus the highest absolute change from summer to winter, although it did not regrow in spring. The rhinal and piriform cortices exhibited a profound winter decline (18.7%) and a clear but less intense spring regrowth (5.4%). We also found a seasonal, reversible (− 13.7%/+13.6%), but sexually dimorphic, pattern in the olfactory bulb, with winter and spring females showing 13.7 and 14.9% lower volumes, respectively. The cerebellum followed a different and more complex pattern. Cerebellar volume was sexually dimorphic in winter animals. Winter females had a 32.2% smaller cerebellum than males, but in spring the females’ cerebellum increased and reached a similar size to males again (Fig. 8; Table 1). The volume of the remaining brain areas measured altogether (“Rest of brain” in Figs. 6, 8; Table 1) followed the pattern of summer-to-winter decrease and the winter-to-spring regrowth. The regrowth was more intense in males than in females, leading to sexual dimorphism in adults.

### Neuron tracing

**Fig. 11:**
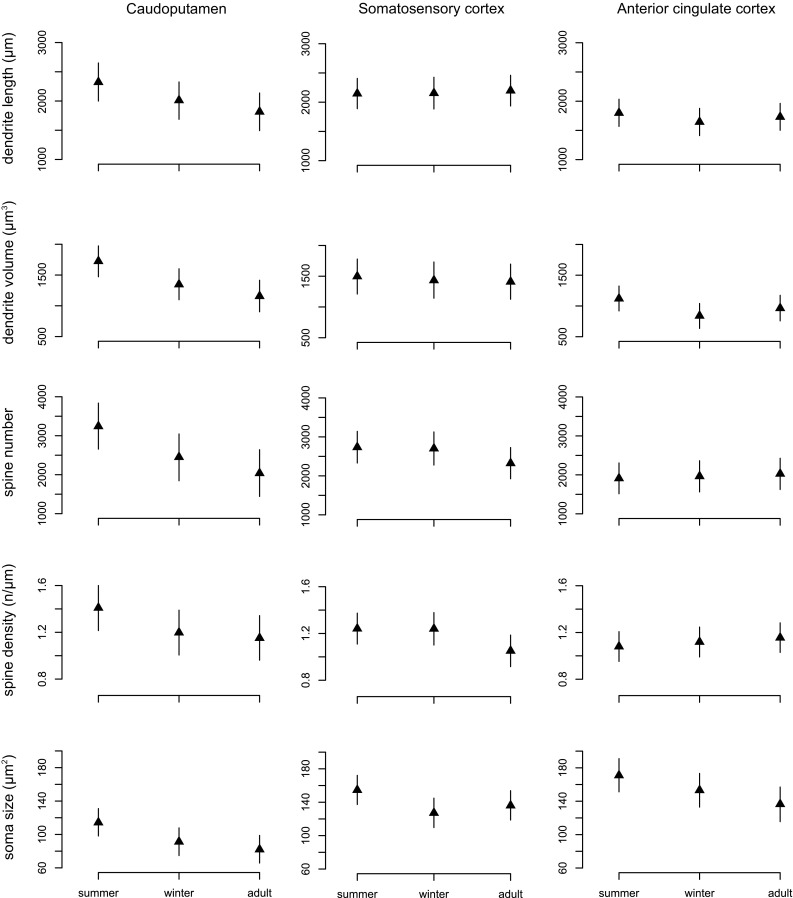
Seasonal changes in dendrite morphology and spine numbers. Graphs depict the results on the median spiny neurons of the caudoputamen (right column) and pyramidal neurons of the somatosensory (central column) and anterior cingulate cortices (right column). Triangles represent the means at each stage (all males) and bars are credible intervals

Our results from the neuron tracings reveal patterns of variation between age groups in the three brain regions we examined (Fig. 11; Table 2). In the caudoputamen, we found a steady decline in all dendritic (21.8% in length and 32.9% in volume), soma (28.1%) and spine (37.1% in spine number and 18.4% in density) measures. This decline was more pronounced in the first phase (summer juvenile to winter subadult) than in the second phase (winter subadult to spring–summer adult) (Table 2). In contrast, the somatosensory cortex only showed a marked decline from summer to winter in soma size (17.7%) and in spine density (15.2%) from winter to adult. We also found a substantial decrease in soma size in the anterior cingulate cortex by 19.9%. In addition, in the anterior cingulate cortex there was a decrease from summer to winter in dendrite volume by 25.0%.

**Table 2:** Mean values derived from neuron morphology during the three age stages, and mean differences between stages and probabilities (*P*) of the difference from summer juveniles to winter subadults (s–w) and from winter subadults to spring–summer adults (w–a) as calculated from the posterior distributions

	Summer juvenile		Winter subadult		Spring–summer adult		Difference summer–winter	*P*(s–w)	Difference winter–adult	*P*(w–a)
	Mean ± SD	95% CrI	Mean ± SD	95% CrI	Mean ± SD	95% CrI				
Somatosensory cortex										
Dendrite length (µm)	2148 ± 418	1882/2409	2155 ± 660	1884/2425	2199 ± 595	1934/2463	7 (0.3%)	0.38	44 (2.0%)	0.46
Dendr. volume (µm^3^)	1498 ± 477	1214/1782	1433 ± 554	1145/1732	1410 ± 483	1127/1700	− 65 (− 4.3%)	0.62	− 23 (− 1.6%)	0.54
Soma size (µm^2^)	155 ± 36	137/172	127 ± 29	109/146	136 ± 32	119/153	− 28 (− 17.7%)	0.99	9 (7.0%)	0.75
Spine number	2736 ± 1057	2332/3134	2703 ± 964	2283/3132	2324 ± 926	1918/2727	− 33 (− 1.2%)	0.46	− 379 (− 14.0%)	0.10
Spine density (n/µm)	1.2 ± 0.3	1.1/1.4	1.2 ± 0.3	1.1/1.4	1.1 ± 0.3	0.9/1.2	< 0.1 (− 0.1%)	0.51	− 0.2 (− 15.2%)	0.97
Cingulate cortex										
Dendrite length (µm)	1799 ± 521	1568/2035	1645 ± 720	1415/1874	1731 ± 470	1498/1959	− 154 (− 8.6%)	0.83	86 (5.2%)	0.70
Dendr. volume (µm^3^)	1120 ± 456	916/1320	841 ± 357	637/1041	965 ± 282	756/1174	− 279 (− 25.0%)	0.97	124 (14.8%)	0.80
Soma size (µm^2^)	171 ± 42	151/191	153 ± 32	133/173	137 ± 23	116/158	− 18 (− 10.4%)	0.89	− 17 (− 10.9%)	0.87
Spine number	1912 ± 689	1519/2307	1965 ± 1365	1562/2366	2028 ± 821	1623/2435	53 (2.8%)	0.57	63 (3.2%)	0.58
Spine density (n/µm)	1.1 ± 0.3	1.0/1.2	1.1 ± 0.4	1.0/1.3	1.2 ± 0.3	1.0/1.3	< 0.1 (3.6%)	0.34	< 0.1 (3.3%)	0.34
Caudoputamen										
Dendrite length (µm)	2324 ± 672	2003/2645	2013 ± 453	1688/2342	1817 ± 549	1494/2137	− 311 (− 13.4%)	0.91	− 196 (− 9.7%)	0.80
Dendr. volume (µm^3^)	1725 ± 543	1472/1978	1348 ± 405	1093/1604	1157 ± 309	899/1412	− 377 (− 21.9%)	0.98	− 191 (− 14.2%)	0.98
Soma size (µm^2^)	114 ± 37	98/131	91 ± 21	74/108	82 ± 9	65/99	− 23 (− 20.2%)	0.97	− 9 (− 10.1%)	0.78
Spine number	3243 ± 1105	2644/3842	2453 ± 930	1859/3060	2038 ± 503	1444/2637	− 790 (− 24.4%)	0.97	− 415 (− 16.9%)	0.83
Spine density (n/µm)	1.4 ± 0.3	1.2/1.6	1.2 ± 0.3	1.0/1.4	1.2 ± 0.2	1.0/1.3	− 0.2 (− 15.0%)	0.94	− 0.05 (− 3.9%)	0.64

## Discussion

### Changes in volume of brain regions

Our results confirm the seasonal pattern of change in the volume of overall brain hemispheres of red-toothed shrews in Southern Germany that was previously reported from Russia and Poland (Bielak and Pucek 1960; Yaskin 1994). However, as expected, the pattern was expressed less strongly: the winter decline was 5.1% less pronounced than in north Poland (Pucek 1965b) and 10.2% less than in the Moscow region (Yaskin 1994). We found a decrease of 16.1% from summer juveniles to winter subadults and a subsequent increase by 9.8% in spring-summer adults. The decline in volume from juveniles to subadults happens in anticipation of winter and hence cannot be seen as an immediate reaction to temperature or food availability, but is more likely genetically encoded. When we analyzed the volume of each brain region separately, we observed that the different brain structures varied in the magnitude of change. Also, in some brain regions, seasonal changes varied between the sexes.

In the mammalian brain, we expect to find positive allometric correlations between overall brain size and each region caused by functional and/or developmental constraints (Finlay and Darlington 1995; Yopak et al. 2010; Charvet et al. 2011). In our results, as the brains varied in size seasonally, each region’s size changed—or remained unchanged—independently of others. This variation might correspond to a mosaic adaptive development, which results in brain structure volumes that dynamically adjust to match the current cognitive demands and energetic constraints of the individuals. For example, summer juveniles would need to meet different cognitive requirements regarding territorial and reproductive behavior as they disperse and compete for territories (Moraleva and Telitzina 1994) than winter subadults, which minimize movement and social interactions to conserve energy. Cognitive demands would be different again in spring adults when shrews expand their home ranges for mate searching (Yaskin 2005; Gonda et al. 2013). This is consistent with our finding that in the winter, shrews underwent a decrease in hippocampal volume followed by regrowth in spring (Fig. 8). In other polygamous species where males show a greater expansion of home range than females, this has been linked to higher performance of males in spatial tests (Gaulin and Fitzgerald 1989; Galea et al. 1996). This is also congruent with the sexual dimorphism we found in the CA1 of adults (Fig. 10), as male common shrews enlarge their ranges more than females do (Stockley et al. 1994; Rychlik 1998; Stockley and Searle 1998). Furthermore, such a functional adaptive explanation is at least partially consistent with the observed decline in cortical regions in the winter. However, the lack of spring regrowth of the neocortex and the fairly extreme seasonal changes in other parts of the brain that are not associated with foraging and social functions, such as the thalamic regions, remain difficult to account for.

Differences in the potential for plasticity between brain structures may also play an important role in constraining the pattern of seasonal variation in regional volumes that can occur. In a mammal with the usual curve of unidirectional brain growth (Dobbing and Sands 1973, 1979), the late developed regions—those where neurogenesis peaks occur later in ontogeny—tend to develop larger since they undergo more rounds of neurogenesis (Finlay and Darlington 1995; Clancy et al. 2001). This could translate into different capacities between regions to undergo plastic changes across seasons. Based on this, we expected the most plastic (latest developed) brain region to reveal the most drastic changes between seasons. However, ontogenetic timing does not seem to determine the intensity of change in our shrews’ brain regions either, as both early (e.g., thalamus) and late developing regions (e.g., neocortex) (Clancy et al. 2001) showed high seasonal variation. Attention should also be drawn to the changes in the cerebellum, which is one of the regions to develop latest in the mammalian brain: the sexual dimorphism in winter, which disappears later in adulthood, seems to be the result of differential timing in development between males and females. Consequently, male shrews reach adult cerebellar size earlier than females in ontogeny (Suárez et al. 1992; Fan et al. 2010; Tiemeier et al. 2011).

Regardless of the pattern of change, energetic limitations are likely to be a primary driver of variability in overall brain size across seasons. Energetic costs of brain computation function and tissue maintenance are extraordinarily high when compared to other physiological processes in different tissues (McNab and Eisenberg 1989; Aiello and Wheeler 1995; Laughlin et al. 1998). This energetic demand is considered an important constraint for development and evolution of brain size (Niven and Laughlin 2008; Bullmore and Sporns 2012). For this reason, the winter decrease in overall brain size of shrews has most commonly been proposed to be a strategy to reduce metabolic consumption during that period (Mezhzherin 1964; Pucek 1970), when food quality is lower (Churchfield et al. 2012) and therefore energy supply becomes a more limiting factor. Consequently, given that different brain structures have different metabolic demands due to their cellular architecture and activity level, we expected those brain regions with the highest metabolic costs to show the most pronounced winter shrinkage. However, such a scenario is unlikely to be the only explanation, based on our results: the magnitude of change of the different regions does not correlate to their metabolic scaling slope (Kaufman 2004; Karbowski 2007). For example, the thalamus which undergoes the strongest seasonal change shows one of the lowest mass-specific metabolic rates among brain regions (Kaufman 2004). Also, it does not explain why some regions do not regrow in the second spring/summer, when food availability is the same for adults and juveniles. Therefore, although energy limitation is probably an important factor to determine the changes in overall brain size, it fails to completely explain the patterns in the different regions.

Although the overall seasonal variation in brain size may be caused by energetic limitation in winter, the variation in the different brain structures appears to be due to a combination of functional adaptations, as well as developmental constraints on plasticity. The size of each brain region is influenced by these factors to different degrees, and these influences may be different in the decrease and regrowth phases of the cycle.

### Variation in neuron size and morphology

Our results on neuron morphology partially supported our expectations in the caudoputamen, but not in the cortical areas. The decline in caudoputamen volume from summer to winter (Fig. 9) was paralleled by a decrease in medium spiny neuron dendrite length and volume, spine number and soma size (Fig. 6; Table 2). These morphological changes resulted in neuronal retraction, which may have contributed to the observed decrease in the volume of the caudoputamen. We found a decrease in dendritic arbor length only in the anterior cingulate cortex. However, together with the decline in soma size in both the anterior cingulate and somatosensory cortices (Fig. 6; Table 2), this is unlikely to explain the − 27.7% volume reduction in the neocortex, of which the somatosensory area makes up a large portion (Catania 2000). One possibility is that greater changes in dendritic trees might be located in other cortical areas, cell layers and/or neuron types. For pyramidal neurons where we assessed the morphology of basal dendrites, part of the seasonal variation in volume could be hidden in the apical dendrites. Also, we must consider alternative mechanisms that affect tissue volume. Because of methodological limitations, we did not quantify axon size and density in this study. In addition, seasonal variation in cell numbers through cell death during autumn and cell recruitment in spring in the olfactory bulbs and dentate gyrus do not substantially contribute to the overall chance in hippocampus mass (Bartkowska et al. 2008). Adult neurogenesis in certain brain regions is a common process in mammals, but the rate of cell proliferation varies between species (Amrein 2015). *Sorex* shrews are an exceptional case with no adult neurogenesis in the dentate gyrus, in contrast with most of mammals (Bartkowska et al. 2008). Other brain regions have not yet been investigated for adult neurogenesis in shrews. But based on previous knowledge, the presence of adult neuron recruitment in other regions except potentially the olfactory bulb is unlikely (Amrein 2015). Volumetric changes are more likely to occur in the neuropil, which makes up the space between cells (Spocter et al. 2012). Finally, mammalian brains show high variation in white matter over the lifetime (Marner et al. 2003). Therefore, future research should assess the seasonal variation in axonal innervation and the energetically costly myelin. If Dehnel’s phenomenon is an energy saving process, myelin would be expected to decrease in winter. Nonexclusively, in a less energetically demanding tissue, we would predict a decrease in the circulatory system to transports nutrients. Thus, we might observe a decrease in the density of microvessels, which may also impact tissue volume (Farkas and Luiten 2001).

Natural processes of neuron shrinkage are not uncommon over the course of ontogeny. During early development of most regions in the vertebrate nervous system, there is a phase of initial overproduction of dendrites and synapses, followed by a period of elimination of surplus connections in an activity-dependent manner (Cowan et al. 1984; Clarke 1990). But this refinement phase often takes place at a perinatal stage in mammals, shortly after birth. In shrews, brain shrinkage is postnatal and lasts seven months, which constitutes half of their life span (Pucek 1970; Churchfield 1990). Dendritic and synaptic elimination do not seem to contribute significantly to the overt volumetric changes that are observed within brain regions in these shrews. There are also abundant instances of brain tissue deterioration in senescent mammals, including humans (Raz et al. 2005). This aging decrease in tissue volume correlates with neuron atrophy, which is caused by a decrease in soma size and in dendritic arbors and spine numbers, both in the neocortex and hippocampus (Geinisman et al. 1978; Anderson and Rutledge 1996; Smith et al. 1999; Dickstein et al. 2007). Such changes in neuron morphology have been linked to a non-pathological decrease in cognitive performance during aging in mammals (Duan et al. 2003; Burke and Barnes 2006). These cellular and cognitive changes might be comparable to the seasonal differences that we found between summer juveniles and winter subadults in brain morphology and cognition (Lázaro et al. 2018). Winter subadults performed more poorly than summer juveniles and adults in a learning test (Lázaro et al. 2018).

The seasonal brain shrinkage and regrowth that we describe result in a dramatic change in size and reorganization of neuroanatomy. Future research on these seasonal brain changes in shrews may lead toward biological and medical applications. The reversibility and rapid remodeling of brain tissue architecture make the common shrew an optimal model for studying possible mechanisms to invert degenerative processes in the nervous system. Furthermore, the connection of brain structure with environmental fluctuations can provide insights into the ecological pressures that shape the development and evolution of the mammalian brain.

## Electronic supplementary material

Below is the link to the electronic supplementary material.

Supplementary material 1 (DOCX 30 KB)
