# Supplementary material for: Profound seasonal changes in brain size and architecture in the common shrew
Source: Brain Struct Funct. 2018 Apr 16;223(6):2823–40. doi: 10.1007/s00429-018-1666-5 (PMC5995987; doi:10.1007/s00429-018-1666-5)
Supplement: Supplementary file 1 — Supplementary material 1 (DOCX 30 KB) [file 429_2018_1666_MOESM1_ESM.docx]

**Supplementary materials for: Profound seasonal changes in brain size and architecture in the common shrew**

Authors: Javier Lázaro^1,2^*, Moritz Hertel^3^, Chet C. Sherwood^4^, Marion Muturi^1,2^, Dina K.N. Dechmann^1,2^

Affiliations: ^1^Max Planck Institute for Ornithology, Department of Migration and Immuno-ecology, 78315 Radolfzell, Germany; ^2^University of Konstanz, Department of Biology, 78457 Konstanz, Germany; ^3^Max Planck Institute for Ornithology, Department of Behavioural Neurobiology, 82319 Seewiesen, Germany.^4^The George Washington University, Department of Anthropology and Center for Advanced Study of Human Paleobiology, 20052 Washington DC, United States.

*Correspondence and lead contact: [jlazaro@orn.mpg.de](mailto:jlazaro@orn.mpg.de)

|  | Summer juvenile | | Winter subadult | | Spring-summer adult | | Difference  summer-winter |  | Difference  winter-adult |  |
| --- | --- | --- | --- | --- | --- | --- | --- | --- | --- | --- |
|  | Mean ± SD | 95% CrI | Mean ± SD | 95% CrI | Mean ± SD | 95% CrI |  | P(s-w) |  | P(w-a) |
| both sexes | 122.9 ± 4.7 | 119.0/127.0 | 102.9 ± 6.4 | 99.1/106.7 | 112.5 ± 7.0 | 108.9/116.0 | -20.0 (-16.3%) | >0.99 | 9.5 (9.3%) | >0.99 |
| males | 122.1 ± 4.6 | 117.0/127.0 | 106.6 ± 2.7 | 101.4/111.7 | 115.6 ± 6.7 | 111.3/120.0 | -15.6 (-12.7%) | >0.99 | 9.1 (8.5%) | >0.99 |
| females | 123.8 ± 5.1 | 118.7/129.0 | 99.9 ± 7.3 | 95.2/104.6 | 108.1 ± 4.9 | 102.9/113.3 | -23.9 (-19.3%) | >0.99 | 8.1 (8.2%) | 0.99 |

**Table S1.** Absolute values for entire hemisphere volumes in the three stages and in both sexes. Values are in mm^3^.

**Table S2.** Corrected volumes for striatal subregions in the three age stages, mean differences between stages and probabilities (P) of the difference from summer juveniles to winter subadults (s-w) and from winter subadults to spring-summer adults (w-a) as calculated from the posterior distributions. The values of each sex are depicted only for the region that revealed sex-specific differences. Volumes are given in µm^3^ and are measured in one hemisphere.

|  |  | Summer juvenile | | Winter subadult | | Spring-summer adult | | Difference summer-winter |  | Difference winter-adult |  |
| --- | --- | --- | --- | --- | --- | --- | --- | --- | --- | --- | --- |
|  |  | Mean ± SD | 95% CrI | Mean ± SD | 95% CrI | Mean ± SD | 95% CrI |  | P(s-w) |  | P(w-a) |
| Nucleus accumbens | | 2.7 ± 0.6 | 2.1/3.2 | 2.6 ± 0.6 | 2.1/3.1 | 2.5 ± 0.4 | 2.1/3.0 | -0.1 (-2.6%) | 0.62 | -0.1 (-2.7%) | 0.38 |
| Amygdala | | 1.5 ± 0.3 | 1.0/2.0 | 1.3 ± 0.2 | 0.8/1.8 | 1.5 ± 0.3 | 1.0/1.9 | -0.3 (-17.0%) | 0.98 | 0.2 (16.9%) | 0.96 |
| Caudoputamen | | 10.7 ± 1.7 | 10.2/11.2 | 8.5 ± 1.1 | 8.0/9.0 | 8.5 ± 0.1 | 8.0/9.0 | -2.2 (-20.7%) | >0.99 | 0.01 (0.1%) | 0.51 |
|  | males | 9.8 ± 0.9 | 9.1/10.5 | 8.2 ± 1.5 | 7.5/8.9 | 8.5 ± 1.0 | 7.9/9.0 | -1.6 (-16.0%) | 0.97 | 0.2 (2.7%) | 0.62 |
|  | females | 11.6 ± 1.9 | 10.9/12.3 | 8.7 ± 0.8 | 8.1/9.3 | 8.6 ± 1.0 | 7.9/9.2 | -2.9 (-25.0%) | >0.99 | -0.2 (-1.7%) | 0.43 |

**Table S3**. Corrected volumes for hippocampal subregions in the three age stages, mean differences between stages and probabilities (P) of the difference from summer juveniles to winter subadults (s-w) and from winter subadults to spring-summer adults (w-a) as calculated from the posterior distributions. The values of each sex are depicted only for the region that revealed sex-specific differences. Volumes are given in µm^3^ and are measured in one hemisphere.

|  | Summer juvenile | | Winter subadult | | Spring-summer adult | | Difference  summer-winter |  | Difference  winter-adult |  |  |
| --- | --- | --- | --- | --- | --- | --- | --- | --- | --- | --- | --- |
|  | Mean ± SD | 95% CrI | Mean ± SD | 95% CrI | Mean ± SD | 95% CrI |  | P(s-w) |  | P(w-a) |  |
| CA1 |  | 6.1 ± 0.9 | 5.7/6.6 | 5.4 ± 0.7 | 5.0/5.8 | 6.1 ± 0.9 | 5.7/6.5 | -0.8 (-12.6%) | 0.97 | 0.7 (13.5%) | 0.97 |
| males | 6.5 ± 0.5 | 5.9/7.0 | 5.6 ± 0.5 | 5.0/6.2 | 6.7 ± 0.6 | 6.2/7.2 | -0.8 (-13.0%) | 0.96 | 1.0 (18.4%) | 0.99 |  |
| females | 5.8 ± 1.2 | 5.2/6.4 | 5.1 ± 0.9 | 4.6/5.7 | 5.3 ± 0.7 | 4.7/5.9 | -0.7 (-11.4%) | 0.92 | 0.1 (2.7%) | 0.62 |  |
| CA2 |  | 2.3 ± 0.3 | 1.9/2.8 | 2.0 ± 0.8 | 1.6/2.4 | 2.4 ± 0.4 | 2.0/2.8 | -0.4 (-15.8%) | 0.94 | 0.5 (24.2%) | 0.98 |
| CA3 |  | 2.5 ± 0.4 | 2.1/2.9 | 2.7 ± 0.4 | 2.3/3.1 | 2.6 ± 0.4 | 2.2/3.0 | 0.2 (8.3%) | 0.13 | -0.1 (-2.6%) | 0.34 |
| Dentate gyrus | | 6.3 ± 0.9 | 5.8/6.7 | 5.3 ± 1.0 | 4.9/5.7 | 6.1 ± 0.9 | 5.7/6.5 | -1.0 (-15.2%) | 0.99 | 0.8 (15.6%) | 0.98 |
| Subbiculum | | 2.2 ± 0.6 | 1.8/2.6 | 2.0 ± 0.4 | 1.6/2.4 | 2.0 ± 0.5 | 1.6/2.4 | -0.2 (-8.5%) | 0.81 | -0.02 (-1.2%) | 0.46 |
